# Describing flares: a mixed methods study comparing patient and clinician perspectives in systemic lupus erythematosus and other rheumatic diseases

**DOI:** 10.1093/rheumatology/keag443

**Published:** 2026-08-20

**Authors:** Martha A Piper, Alice Tunks, James A Bourgeois, Lucy Calderwood, David D’Cruz, Alessandra Bortoluzzi, Arjoon Arunasalam, Shaista Tayabali, Sydnae Taylor, Pratyasha Saha, Max Yates, Paige Hamilton-Conaty, Arvind Kaul, Melanie Sloan

**Affiliations:** Department of Psychology, University of London, Goldsmiths, UK; Department of Psychology, University of Surrey, Guildford, UK; Department of Psychiatry and Behavioral Sciences, University of California Davis Medical Center, Sacramento, USA; Patient Partners, UK; The Louise Coote Lupus Unit, Guy’s and St Thomas’ Hospitals NHS Foundation Trust, London, UK; Rheumatology Unit, Department of Medical Sciences, University of Ferrara and Azienda, Ospedaliero-Universitaria S. Anna, Ferrara, Italy; Institute of Psychiatry, Psychology and Neuroscience, King’s College London, and South London and Maudsley NHS Foundation Trust, London, UK; Patient Partners, UK; Primary Care Unit, Department of Public Health and Primary Care, University of Cambridge, Cambridge, UK; Faculty of Medicine and Health, University of East Anglia, Norwich, UK; Faculty of Medicine and Health, University of East Anglia, Norwich, UK; School of Medicine, University of California Davis Medical Center, Sacramento, USA; Centre for Rheumatology, St Georges’ Hospital, London, UK; Primary Care Unit, Department of Public Health and Primary Care, University of Cambridge, Cambridge, UK; Faculty of Medicine and Health, University of East Anglia, Norwich, UK

**Keywords:** rheumatology, flare, patient perspective, clinician perspective, recognition, management, patient and public partnership, systemic lupus erythematosus

## Abstract

**Objectives:**

Systemic autoimmune rheumatic diseases (SARDs) relapse and remit, with periods of increased disease activity called ‘flares’. This study compared patient and clinician perspectives of flares.

**Methods:**

Mixed-methods approach combining an international co-produced survey and in-depth interviews. SLE patients and clinicians rated statements about flares on Likert scales from ‘*never*’ to ‘*always*’. Quantitative data were analysed using *t* tests and ANOVA. Patient and clinician interviews were analysed thematically. Quantitative and qualitative analyses were triangulated by exploring converging, diverging and explanatory findings across the datasets.

**Results:**

Qualitative (*N* = 31 SARD patients, *N* = 12 clinicians) and quantitative (*N* = 443 SLE patients, *N* = 258 clinicians) results indicated differential patient and clinician perspectives. The main themes identified were: characteristics/consequences of flares; recognition of flares; and treatment for flares (medical and/or self-management). Patients and clinicians had significantly different mean ratings for 12/17 flare statements. Notably, regarding flare onset, 39% of patients compared with 14% of clinicians said flares ‘*often*’ or ‘*always*’ started within minutes or hours (*P* < 0.001). We found differences in identification of flares, with patients rating ‘*patient can tell*’ significantly higher than clinicians (*P* < .001), including awareness of prodromal symptoms. Clinicians were significantly more likely to think flares required increased (*P* = 0.005) or new (*P* < .001) medication. There was consensus about the impact of flares on wellbeing.

**Conclusion:**

We highlight differences in how SARD patients and clinicians describe flares, notably related to onset, recognition and intervention. These differences challenge the prevailing reliance on clinician-derived disease activity measures and biomarkers for flare assessment, highlighting the need for integration of patient and clinician perspectives.

Rheumatology key messages

- Patients and clinicians perceive SLE flares differently, particularly regarding the speed of onset, recognition and treatment requirements.
- Generic disease and flare activity measures are unlikely to capture each individual’s unique flare characteristics.
- Effective flare management requires validating patient experiences, improved communication and adaptable individualised care plans.

## Introduction

Symptoms of systemic autoimmune rheumatic diseases (SARDs) are wide-ranging and can affect physical, cognitive and emotional functioning [1]. SARDs symptoms can relapse and remit, with periods of increased disease activity often referred to as flares. The word ‘flare’ is not well defined in research, with studies using a variety of classifications, including thresholds on disease activity indices, changes in medical treatment and physician and patient assessments [2, 3]. In systemic lupus erythematosus (SLE), many clinicians rely on objective changes in disease activity defined by disease activity indices. Many of these do not include the patient perspective [4, 5]. Furthermore, measures which do capture the patient perspective, such as the LFA-REAL™ system for SLE, have shown only modest correlations with clinician-based measures, highlighting potential areas of discordance between patient experience and clinical assessment [6].

Qualitative research has found that SLE patients describe flares in terms of symptoms, whereas rheumatologists define flares based on their assessment of disease activity, with over half requiring objective findings such as changes in test results [7]. In our team’s previous research about incipient flares, clinicians ranked the patient’s view of whether their disease was flaring the lowest, below the clinicians’ own assessment and diagnostic laboratory test results [8]. A special interest group consisting of mainly clinical researchers defined rheumatoid arthritis (RA) flares as a worsening of disease activity, with persistent and enduring symptoms, leading to initiation or change in therapy [9]. However, research shows that RA patients do not view the initiation of changes in immunomodulation treatment as an essential aspect of flares [10]. Research into juvenile spondylarthritis has identified that these differences in perspective may be driven by patients having specific insights into pain, wellbeing and daily function, to which clinicians are not privy [11]. Therefore, differences in how patients and clinicians use the term flare may be related to its purpose; patients use ‘flare’ to make sense of and communicate symptoms to those around them, such as in work or social contexts, whereas clinicians use it for triage and treatment purposes.

The absence of patient perspectives in flare assessments is concerning, given the unique insights patients often have into impending flares. Studies show that SLE patients recognise the warning signs of flares, known as prodromes, which can include subclinical neuropsychiatric symptoms such as nightmares and sensory symptoms [12]. These prodromal symptoms vary among patients but have high intra-individual consistency. This allows patients to identify patterns of incipient flares over time which, if communicated to clinicians, could lead to earlier identification, validation and potential treatment modification.

Some recent efforts have been made to integrate both patient and clinician views of flares into diagnostic measures. For example, the FLARE questionnaire for identifying RA flares includes aspects most important to patients (daily functioning, symptoms and wellbeing) and those prioritised by clinicians (joint swelling and pain, patient global assessment and CRP level) [13]. Other common measures for RA disease activity, such as Disease Activity Score (DAS-28) and Simplified and Clinical Disease Activity Indices (SDAI and CDAI), use the absolute change in score to measure whether or not a patient is in a flare state [14]. However, thematic analysis has revealed unique patient insights and variation in flares, as well as the multi-dimensional impact of flares on patients’ lives, which may not be obvious in clinical consultations or captured using rigid score-based definitions [3].

It is evident that there is a need to consider both patient and clinician views of flares. This study aims to understand and compare the key aspects of flares from the perspectives of SARDs patients and clinicians.

## Methods

### INSPIRE research programme

This study (‘INSPIRE-Flare’) is part of the second stage of the INSPIRE (**I**nvestigating **N**europsychiatric **S**ymptom **P**revalence and **I**mpact in **R**heumatology patient **E**xperiences) research programme. The initial INSPIRE research incorporated inter-linked studies exploring different aspects of neuropsychiatric symptoms. INSPIRE-Flare was conceptualised and prioritised following concerns arising during INSPIRE that patient experiences of flares were given lower priority than clinician assessments and objective markers. INSPIRE-Flare workstreams include the data reported here on perspectives of flares, and a related study exploring patient views of triggers and preventers of their flares.

### Design

Qualitative data were collected via semi-structured interviews exploring patient and clinician perspectives on flares and open-ended responses about flares from patients in the INSPIRE-Flare survey. Quantitative data from patients and clinicians were collected via the INSPIRE-Flare surveys. To provide specificity for clinicians, and ensure comparability between patients and clinicians responses, we asked clinicians about their perspectives on flares in SLE and compared this with SLE patients’ views only. SLE was selected as an example of how clinician and patient perspectives compare quantitatively. We used interview data from all SARD patient groups to highlight the similarities in flare experiences, although we will report on and compare survey responses from other disease groups in a subsequent paper. The survey and interviews were conducted simultaneously and the datasets were analysed independently, with neither informing the other. Quantitative and qualitative results were triangulated as outlined in the Triangulation section below. For more information on study design and other aspects of methods, see Supplementary Data S1.

### Patient and public partnership

Patients and clinician research partners were involved as equal members of our research team at every stage of this study to increase the relevance and impact of our findings. We used the GRIPP2 checklist to monitor partnership (see Supplementary Data S1).

### Materials

The interview topic guide asked how patients and clinicians described flares, what happened during flares, visibility and identification of flares, medication changes and self-management for flares.

The INSPIRE-Flare surveys included a list of 17 statements about flares which patients and clinicians responded to on a Likert scale of ‘*always*’, ‘*often*’, ‘*sometimes*’, ‘*rarely*’ and ‘*never*’. See Table 1 for the statements and labels for data presentation. Patients added additional comments about what a flare meant to them in an open-text response question.

**Table 1:** Shortened and neutralised flare statement labels.

Patient survey statement	Clinician survey statement	Statement label
Flares involve the emergence of new disease symptoms		New symptoms emerge
Flares occur after a period of disease stability or remission		Following disease stability
Flares involve changes in clinical test results (e.g. blood or urine tests)		Changes in clinical tests
Flares start suddenly (in a matter of min or h)		Start suddenly
Flares start gradually (in a matter of days)		Start gradually
Flares start very gradually (in a matter of weeks or months)		Start very gradually
Flares last for a matter of days		Last for days
Flares last for a matter of weeks		Last for weeks
Flares last for a matter of months		Last for months
Flares involve a worsening of disease symptoms compared with when I’m well	Flares involve a worsening of disease symptoms compared with when the patient is well	Symptoms worsen
Flare symptoms are similar each time	Flare symptoms are similar each time for the same patient	Similar symptoms each time
Flares require an increased dose of prescribed medication (independent of whether you receive it)	Flares require an increased dose of prescribed medication	Require increase in medication
Flares require prescription of new medication (independent of whether you receive it)	Flares require prescription of new medication	Require new medication
Flare symptoms affect my quality of life	Flare symptoms affect patient quality of life	Affect quality of life
I know when I am flaring	A patient will know if their lupus is flaring	Patient can tell
Doctors can tell if I am flaring by physical examination	The patient’s clinician will be able to tell if the lupus is flaring by physical examination	Doctor can tell
There are symptoms/signs that come before a flare, which are a ‘bad sign’ that a flare is coming (this is known as a prodrome)	The patient will have symptoms that are a ‘prodrome’ before a flare that indicates the imminent onset of a flare	Prodromes of flares

### Participants

All participants had to be aged 18 or older and able to read and write in English. All patients self-reported a diagnosis of a SARD on clinical correspondence and all clinicians self-reported having experience of working with SARDs patients in clinic. We recruited for the patient survey through a poster which was disseminated through autoimmune disease charities, social media, online forums, community programs and selected NHS rheumatology clinics in England. We also invited previous INSPIRE study participants who had consented to receive information about future studies via email. We recruited for the clinician survey through social media and the research team’s professional networks. Patient and clinician interviewees were recruited through emailing survey respondents from the team’s previous studies who had opted in to being invited to interview. Patients and clinicians were purposively sampled to include a range of demographics and disease experiences for online interviews via Microsoft Teams. Based on the model of informational power, sample size was evaluated iteratively according to the research question, heterogeneity in participants’ disease type and the depth of the interviews and thematic analysis [15]. A final sample of 43 (31 patients and 12 clinicians) was deemed sufficient. The surveys were completed in Qualtrics.

### Procedure

Ethical approval was obtained through the Cambridge Psychology Ethics Committee: PRE.2025.009 on 14 February 2025 (patients) and PRE2024.062 (clinicians) and the Health Research Authority: IRAS ID 351763 on 12 February 2025. All participants were given the participant information sheet and provided informed consent in Qualtrics before completing the survey and verbally before interviews.

### Analysis

#### Qualitative

Interviews were transcribed verbatim, imported into NVivo and analysed thematically by the lead researcher [16]. This included immersion in the data, coding and development of preliminary themes. Data collection and coding were conducted iteratively, with the researcher regularly reviewing the interview data already gathered and this informing the focus and number of subsequent interviews. Themes were developed iteratively and refined with involvement from patient partners, clinicians and researchers. Illustrative quotes were selected to represent themes and subthemes.

#### Quantitative

Likert scale responses were assigned numerical values for statistical analysis (0 = Never, 1 = Rarely, 2 = Sometimes, 3 = Often, 4 = Always). *T* tests and analyses of variance (ANOVA) were performed in SPSS to investigate associations between variables of interest according to a pre-agreed statistical analysis plan.

#### Triangulation

Qualitative and quantitative data were collected simultaneously, with neither informing the other. Thematic and statistical analyses were conducted separately and the results were combined accordingly to the triangulation protocol [17]. This included consideration of areas of convergence and divergence between the two data sources, which were analysed and discussed by multiple co-authors including the lead and senior authors and patient co-investigators. Themes and subthemes were also checked against open-ended survey responses as a form of confirmatory data triangulation.

#### Research team and reflexivity

All interviewers were female, but there was variety in interviewer age, ethnicity and professional seniority. One interviewer had personal experience of living with a SARD. Interviewers reflected on their own biases, expectations and experiences and how this influenced their interview style and interpretation of the data. Interviewer bias was managed by ensuring all questions were clear and open-ended and through discussions with the multi-disciplinary team.

## Results

Of the *N* = 443 SLE patients completing the survey, 95% were female and 75% were White (Table 2). Clinician survey respondents (*N* = 258) were also predominantly female (68%) and White (62%). The majority worked in rheumatology (66%).

**Table 2:** Participant demographics.

	Semi-structured interviews				INSPIRE-Flare survey			
	SARD patients (N = 31)		Clinicians (N = 12)		SLE patients (N = 443)		Clinicians (N = 258)	
Characteristic	Count	%	Count	%	Count	%	Count	%
**Age**								
18–29	4	12.9%	0	0.0%	30	6.8%	6	2.3%
30–39	5	16.1%	4	33.3%	71	16.0%	73	28.3%
40–49	3	9.7%	3	25.0%	103	23.3%	78	30.2%
50–59	9	29.0%	1	8.3%	118	26.6%	58	22.5%
60+	10	32.3%	4	33.3%	120	27.1%	43	16.7%
Missing data	0	0.0%	0	0.0%	1	0.2%	0	0.0%
**Gender**								
Female	25	80.6%	6	50.0%	420	94.8%	176	68.2%
Male	6	19.4%	6	50.0%	21	4.7%	82	31.8%
Transgender	0	0.0%	0	0.0%	1	0.2%	0	0.0%
Missing data	0	0.0%	0	0.0%	1	0.2%	0	0.0%
**Ethnicity**								
Asian	2	6.5%	2	16.7%	50	11.3%	30	11.6%
Black or African	1	3.2%	0	0.0%	24	5.4%	17	6.6%
Hispanic or Latino/a/x	3	9.7%	0	0.0%	10	2.3%	38	14.7%
Middle Eastern or North African (MENA)	0	0.0%	0	0.0%	3	0.7%	2	0.8%
Mixed or Multiple Ethnicities	2	6.5%	0	0.0%	14	3.2%	2	0.8%
White	23	74.2%	10	83.3%	331	74.7%	160	62.0%
Other	0	0.0%	0	0.0%	11	2.5%	9	3.5%
**Disease**								
IA	1	3.2%			0	0.0%		
Multiple primary	8	25.8%			0	0.0%		
Myositis	2	6.5%			0	0.0%		
PMR	1	3.2%			0	0.0%		
RA	5	16.1%			0	0.0%		
Sjögren’s disease	5	16.1%			0	0.0%		
SLE	4	12.9%			443	100.0%		
Systemic sclerosis (SSc)	2	6.5%			0	0.0%		
Vasculitis	3	9.7%			0	0.0%		
**Speciality**								
Rheumatology			8	66.7%			169	65.5%
Primary care (GP)			2	16.7%			29	11.2%
Obstetrics/Gynaecology			0	0.0%			28	10.9%
Internal medicine			0	0.0%			12	4.7%
Psychiatry			1	8.3%			8	3.1%
Other			1	8.3%			12	4.7%

**Figure 1:**
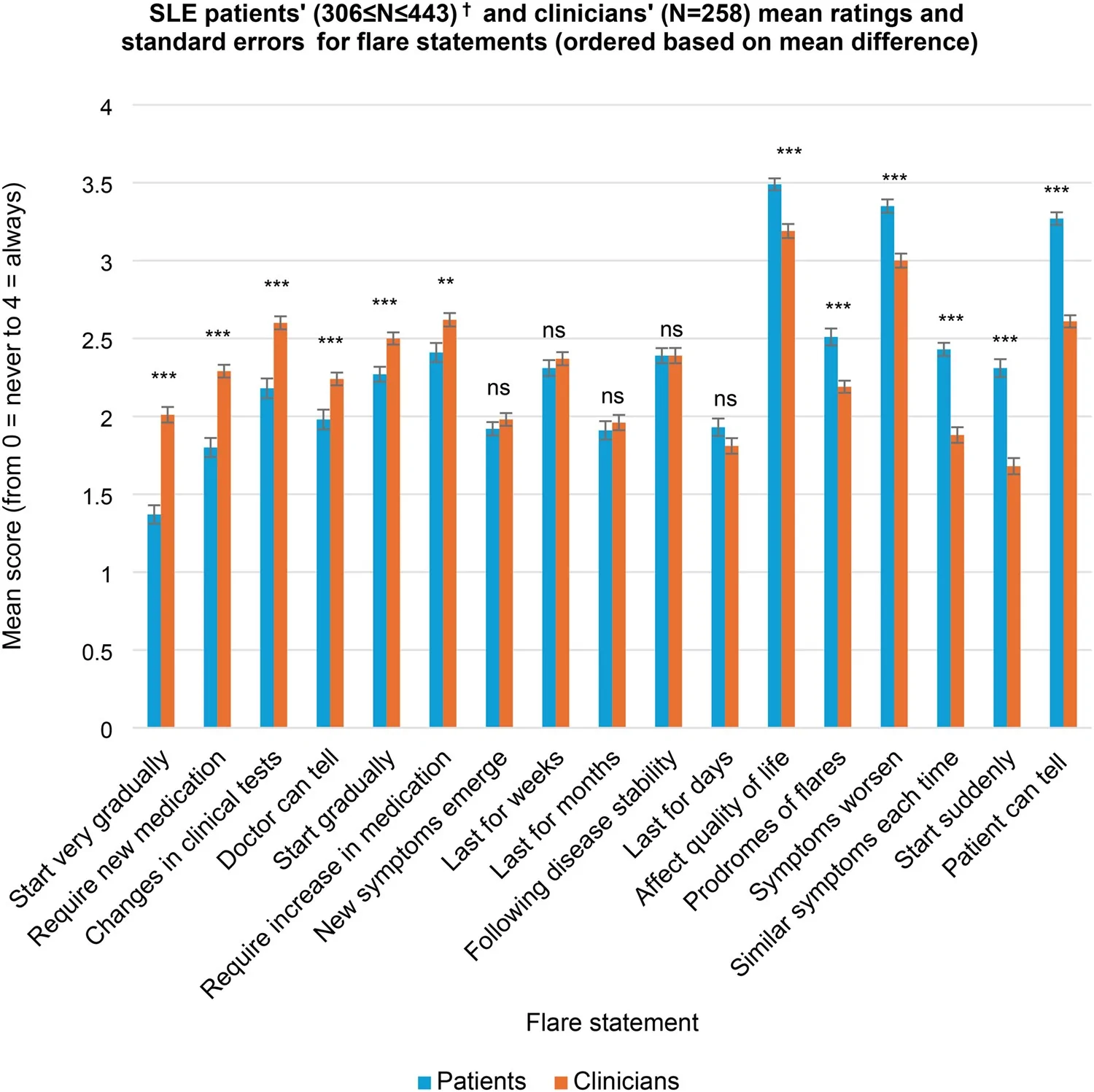
Comparison of SLE patients’ and clinicians’ mean ratings of SLE flare statements. Note: *T* test significance indicated by index: ***, *P* < 0.001; **, *P* < 0.01; *, *P* < 0.05; and ns, non-significant at *P* > 0.05. †N varies for each statement as it excludes patients selecting ‘not sure’

SLE patient and clinician mean ratings were significantly different for 12 of the 17 statements (Fig. 1). The statement which had the highest patient rating relative to clinician rating was ‘*patient can tell*’ (mean difference = 0.66, standard error = 0.06, *P *< 0.001). The statement which had the highest clinician rating relative to patient rating, and was also the lowest rated by patients overall, was ‘*start very gradually*’ (mean difference = 0.64, standard error = 0.06, *P *< 0.001). The statement rated lowest by clinicians was ‘*start suddenly*’, revealing a key difference in patients’ and clinicians’ perceptions of the speed of flare onset.

### Characteristics and consequences of flares

#### A spectrum of worsening symptoms and the dynamics of flare development

The most discussed aspect of flares by both clinicians and patients was the worsening of disease symptoms:

‘*A flare might hit them very dramatically, very briefly, but very different, so … others it’s more gradual onset, which makes the definition of when it starts a bit a bit less clear*’ (C1, psychiatrist, 60–69)

‘*It’s literally all my symptoms coming back within three days and I’m ill again*.’ (P10, myositis, 50-59)

**Figure 2:**
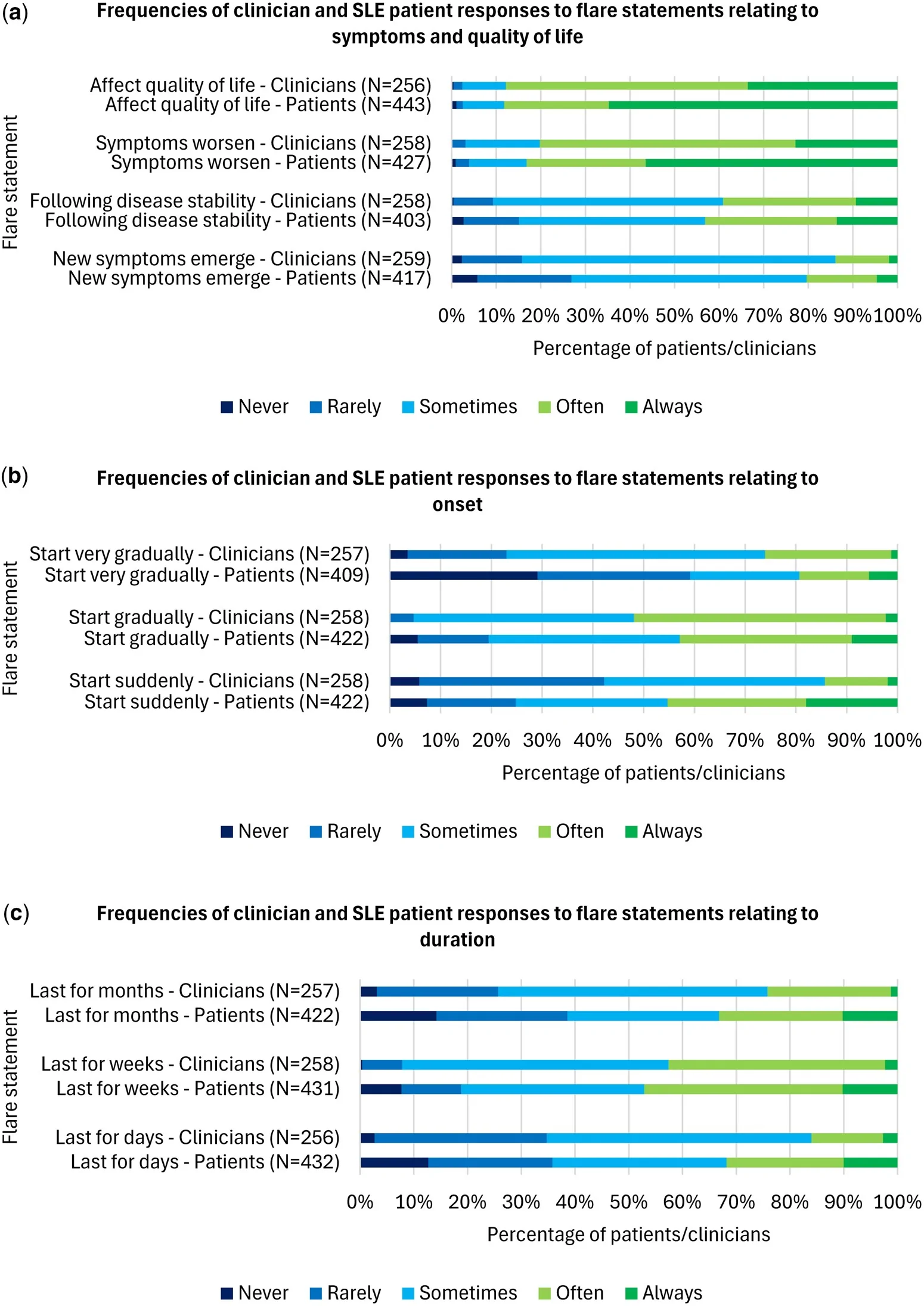
Frequencies of clinician and SLE patient responses to flare statements relating to symptoms and quality of life. (**A**) Relating to symptoms and quality of life. (**B**) Relating to onset. (**C**) Relating to duration

This was mirrored in the survey; 83% of SLE patients and 80% of clinicians said that symptoms ‘*often*’ or ‘*always*’ worsened during SLE flares (Fig. 2A).

The severity of flare symptoms varied, with the majority of patients describing extreme, debilitating symptoms. For example, one patient rated their pain a ‘*15 out of 10*’ (P21, multiple primary, 40–49). Other patients described more minor changes:

‘*If you say flare, I think of a big firework going off or something like that. What I experienced is like, I don’t know a pop, you know? … not even a small banger.*’ (P30, RA, 70+)

Patients and clinicians gave examples of symptoms which worsen during flares, with ‘*pain*’, ‘*fatigue*’ and ‘*aches*’ (multiple participants) being common. Some participants also described cognitive symptoms: ‘*a harder time talking, expressing myself*’ (P4, Sjögren’s disease, 50–59), mental health symptoms: ‘*I feel like dying. I don’t want to be here anymore*’ (P18, multiple primary, 50–59) and sensory changes.

There were significant differences between SLE patients’ and clinicians’ survey responses to statements relating to flare onset, with 45% of patients compared with 14% of clinicians saying flares ‘*often*’ or ‘*always*’ started suddenly (in a matter of minutes or hours) (mean difference = 0.63, CI = [0.477,0.78], *P* < 0.001) (Fig. 2B). Conversely, clinicians rated ‘*start very gradually (in a matter of weeks or months)*’ highest out of the onset statements, indicating a key discrepancy between patients’ and clinicians’ views of flare onset.

Interview data revealed variation in how patients described the onset of flares, with some describing a ‘*sudden, painful incident that’s temporary*’ (P14, RA, 70+) and others saying, ‘*it’s a bit of a gradual thing for me, it’s not, oh I felt alright yesterday, and I feel like absolute death today*’ (P5, Sjögren’s disease, 70+). Clinicians acknowledged this patient-to-patient variation:

‘*A flare might hit them very dramatically, very briefly, but very different, so … others it’s more gradual onset, which makes the definition of when it starts a bit a bit less clear*’ (C1, psychiatrist, 60–69)

There were no significant differences between SLE patients’ and clinicians’ survey responses to statements relating to flare duration (Fig. 2C).

#### Flares impact daily life

Many patients defined flares in terms of the repercussions they had on their daily life. Examples included having to ‘*miss quite a lot of school due to pain flares*’ (P3, multiple primary, 30–39) and ‘*tasks will become difficult, sort of getting up out of a chair, climbing the stairs and bending over*’. (P25, myositis, 50–59):

‘*When you have a flare up with an autoimmune condition, it literally leaves you bed bound and paralysed … I’ve had my mum bathe me, my dad dress me up, my close friends come and cook for me and clean my house*’ (P18, multiple primary, 50–59)

This was corroborated in the survey, where the statement rated highest by patients and clinicians was flares ‘*affect quality of life*’, with 88% of both groups responding ‘*often*’ or ‘*always*’ (Figs 1 and 2A). Patients’ mean rating was significantly higher than clinicians’ (mean difference = 0.30, standard error = 0.07, *P* < 0.001) (Fig. 1).

Patients used words such as ‘*frightening*’ (P14, RA, 70+) and ‘*horrendous*’ (P13, SLE, 30–39) to evoke the fear associated with flares. Others described how flares were ‘*looming over*’ them (P10, myositis, 50–59) and explained how flares made them ‘*surrender again*’ and ‘*start back*’ at the beginning (P8, vasculitis, 50–59).

Some clinicians considered the effects of flares on patients’ quality of life, emphasising the importance of treating patients holistically:

‘*Checking that people are, you know, functioning in society and you know checking are they managing, if they’re at work, are they managing?*’ (C8, rheumatologist, 60–69)

However, clinicians generally used more clinical language to describe flares, such as ‘*stereotypic, discrete … episodic meaning sometimes it’s there and sometimes it’s not*’ (C1, psychiatrist, 60–69), ‘*significant change from the, let’s say, baseline status*’ (C3, rheumatologist, 40–49) or ‘*an exacerbation*’ (C7, rheumatologist, 40–49).

### Recognition of flares

#### Difficulty distinguishing flares from baseline disease

Some clinicians expressed difficulty recognising flares and distinguishing them from the baseline level of disease activity or symptoms:

‘*When does the worsening symptom cross the line to become a separate little episode from the pain* [the patient] *had a week ago?*’ (C1, psychiatrist, 60–69)

A minority of patients also cited difficulties identifying flares, describing them as ‘*not anything very clear-cut*’ (P26, vasculitis, 60–69). For some patients, this was related to the ever-changing nature of their symptoms, which meant what they considered a baseline, non-flare state was constantly shifting.

#### Patients know their flares best

Many patients reported that they could tell when their flares were coming, noticing symptoms such as ‘*increased fatigue could be an indication*’ (P15, SLE, 30–39) and ‘*ulcers in my mouth then it goes downhill from there*’ (P22, multiple primary, 40–49).

**Figure 3:**
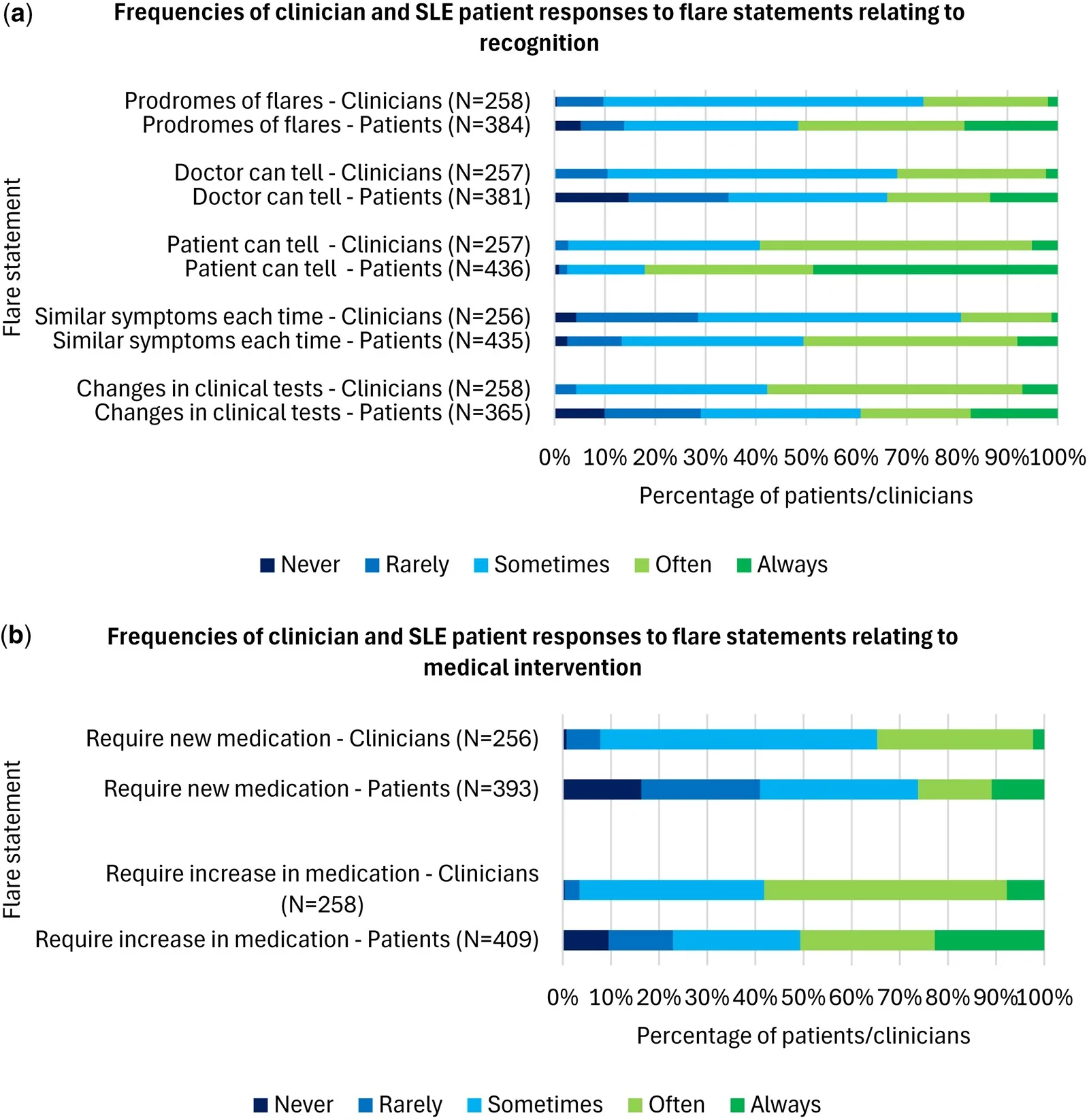
Frequencies of clinician and SLE patient responses to flare statements relating to recognition. (**A**) Relating to recognition. (**B**) Relating to medical intervention

Across all statements in the survey, patients’ and clinicians’ mean ratings differed most for the statement ‘*patients can tell*’ (mean difference = 0.66, standard error = 0.06, *P* < 0.001; Fig. 1), with 82% of patients compared with 59% of clinicians selecting ‘*often*’ or ‘*always*’ (Fig. 3A).

In contrast to survey respondents, many clinician interviewees recognised the importance of patient insight into flares, saying ‘*we need to really listen carefully to the patient*’ (C3, rheumatologist, 40–49) and ‘*the patients often know, like, I’m about to flare*’ (C10, GP, 30–39). They viewed patient vigilance as a key part of flare prevention and management, describing it as a collaborative effort:

‘*Your vigilance to the flares, that’s part of good self-management and I want to kind of help you with that, but this is how you can help you – this is how you could help you to help me to help you*’ (C1, psychiatrist, 60–69)

Some noted that the word flare ‘*means different things to different patients*’ (C2, rheumatologist, 50–59) and therefore patient understanding of their own flare patterns was crucial if flares were to be treated quickly and effectively.

#### Clinically invisible flares and reliance on blood tests

Both patients and clinicians spoke about some flares being clinically invisible; in other words, not being identifiable through blood tests or physical examination. This presented challenges for both patients and clinicians.

On being told their blood tests were normal, many patients questioned whether their symptoms were real, which could make them feel invalidated:

‘*My tests were normal and it’s like, but I’m not normal. Something bad is going on with me. And so that took a real hit on my self-esteem.*’ (P4, Sjögren’s disease, 50–59)

This was reflected in the quantitative analysis, as clinicians rated ‘*doctors can tell*’ and ‘*changes in clinical tests*’ significantly higher than patients (mean difference = −0.26, CI = [−0.407, −0.11], *P *< 0.001 and mean difference = −0.42, CI = [−0.579, −0.279], *P *< 0.001, respectively). Patients rated ‘*similar symptoms each time*’ and ‘*prodromes of flares*’ significantly higher than clinicians (mean difference = 0.55, CI = [0.425,0.680], *P* < 0.001 and mean difference = 0.32, CI = [0.194,0.455], *P *< 0.001, respectively).

In interviews, clinicians acknowledged that flares were not always visible on physical examination or validated by diagnostic laboratory tests, with one rheumatologist calling this ‘*lying blood phenomenon whereby they have worsening symptoms, and their bloods are always within the normal range*’ (C5, rheumatologist, 40–49).

A minority of clinicians explained that they would be less likely to provide medical treatment for flares if there were no visible changes, saying they were ‘*more inclined to intervene if I see those changes*’ rather than a ‘*massive mismatch* [between patient reports and test results]’ (C4, rheumatologist, 30–39). One clinician discussed only attributing common symptoms, such as fatigue, directly to SLE if blood tests indicated active disease:

‘*You get to the point where if all the markers of lupus for that person have disappeared, the fatigue must be due to something else.*’ (C11, rheumatologist, 60–69)

Other clinicians emphasised the problems with relying too heavily on blood tests, describing recognition of autoimmune symptoms as ‘*an art*’ (C12, rheumatologist, 60–69) that required instinct and experience:

‘*Symptoms outweigh everything else … the blood tests can act as a guide, but history and physical examination far outweigh any blood tests we use, for me.*’ (C12, rheumatologist, 60–69)

### Treatment of flares: medical intervention, self-management or both?

#### Medical intervention perceived as a necessary characteristic of flares by clinicians

While medical intervention was infrequently cited by patients as central to flare identification, clinicians commonly emphasised its role as a defining feature of disease flares and a determinant of treatment adjustment. This included: ‘*a slight increase to baseline medication*’ or ‘*a different medication for the period of the flare*’ (C1, psychiatrist, 60–69):

‘[A flare is]* something that probably I cannot manage with not (sic) pharmacological interventions. And so, I want to step up or change the medications.*’ (C3, rheumatologist, 40–49)

This was reflected in the survey, where clinicians rated statements about flares requiring medical intervention significantly higher than patients, specifically ‘*require increase in medication*’ (mean difference = −0.21, CI = [−0.359, −0.065], *P *= 0.005) and ‘*require new medication*’ (mean difference = −0.49, CI = [−0.631, −0.342], *P *< 0.001) (Fig. 1). A total of 35% of clinicians said flares ‘*often*’ or ‘*always*’ required new medication, compared with 26% of patients (Fig. 3B).

Some clinicians spoke about needing to intervene not only to treat the flare but to prevent future flares happening by controlling the disease more effectively long-term:

‘*The important thing is that you change the longer-term treatment as well. Otherwise, when the* [cortico]*steroids wear off, they’ll just be kind of back to where they started.*’ (C7, rheumatologist, 40–49)

#### Self-management by necessity: systemic constraints and clinical caution

Clinicians and patients acknowledged barriers to medical intervention for flares due to difficulties accessing appointments. Patients described having ‘*nobody to ask’* (P5, Sjögren’s disease, 70+) and long wait times for appointments: ‘*You have to be able to do it yourself more now* [after the COVID-19 pandemic], *because that help isn’t there*’ (P16, multiple primary, 50–59).

Clinicians also discussed slow response times, long wait lists and challenges communicating across specialities. The impact of this was that patients only reported or sought medical help for their most severe flares; therefore, introducing a sampling bias in terms of clinicians’ perspectives of how patients typically experienced flares.

These challenges were framed within a broader critique of the healthcare system, which clinicians described as inefficient and time poor. As a result, self-management was often encouraged as a practical first option for flares:

‘*I think that they need to self-manage first … It’s not a service that’s completely functional in most departments across the UK, there are waiting times, and I think in all reality majority of people’s flares settle by the time that they get the return call.*’ (C2, rheumatologist, 50–59)

Patients reported that when they did get appointments, clinicians could be reticent to prescribe what patients thought they needed for effective disease control:

‘*I know what I need … but it’s whether they’re willing to give you what you need*’ (P16, multiple primary, 50–59)

Often this was reported to be related to an unwillingness of clinicians to prescribe aggressive treatment for patient-reported symptoms without corresponding objective evidence. Clinicians reflected on this too, explaining they often felt flares hadn’t progressed to the stage where medical intervention was indicated:

‘*You don’t want to react too soon and then suddenly whack them on a strong medication and then they get a chest infection and end up in hospital*’ (C7, rheumatologist, 40–49)

Some patients spoke about self-medicating if they felt they needed to get around the issues of clinicians’ unavailability and unwillingness:

‘*Would I just put the dose on and then just say I’ve mislaid, you know, I’ve mislaid the patch so therefore I need another one? I probably would if I felt I got that barrier.*’ (P15, SLE, 30–39)

#### Self-management learnt through experience and complementary to medical treatment

Patients described gaining a deeper understanding of their condition through lived experience, which helped them identify what worked for their bodies, how their medications responded and when flares might be approaching:

‘*You start to really understand … you gain confidence over time and also your doctor tends to trust you a little bit more*’ (P15, SLE, 30–39)

Clinicians reported actively supporting this learning process, encouraging patients to become familiar with their disease and engage in self-management strategies:

‘*The better a patient knows their disease and the better we know their disease, that does make a big difference in terms of when intervention happens*’ (C4, rheumatologist, 30–39)

Faced with barriers to timely or effective treatment, patients often became confident in methods of self-management. Strategies included ‘*slow down*’ (P12, multiple primary, 40–49), ‘*sleep*’ (P4, Sjögren’s disease, 50–59), ‘*breathing, mindfulness*’ (C6, physiotherapist, 30–39), ‘*gentle exercise and stretching*’ (C4, rheumatologist, 30–39) and ‘*psychological support*’ (C8, rheumatologist, 60–69). Other patients described just having to ‘*ride it out*’ (P13, SLE, 30–39). When discussing pacing and resting, patients described *‘frustration, because you want to still do it*’ (P26, vasculitis, 60–69) and some said they ended up *‘making myself worse trying to be superwoman*’ (P13, SLE, 30–39).

Some patients preferred self-management due to past experiences with ineffective treatments or wanting to avoid additional medications or ‘*wasting people’s time*’ (P13, SLE, 30–39). Others attributed their approach to personal traits, such as stoicism or pain tolerance:

‘*I’m not really a person that complains a lot or you know, I’m really strong about pain. I just take pain very well, so I usually go with it.*’ (P17, Sjögren’s disease, 18–29)

Importantly, both patients and clinicians recognised that neither medication nor self-management alone was sufficient:

‘*Medications will not do the whole job … there is need for a lifestyle that includes the management of chronic condition*’ (C3, rheumatologist, 40–49).

## Discussion

Triangulation of quantitative and qualitative findings demonstrated evidence of multiple differences between patient and clinician perceptions of SLE flares. Significant differences between patient and clinician ratings were found for 12/17 statements related to flares. Key areas of divergence between these perspectives include the speed of flare onset, the methods used for flare recognition and whether flares necessitate medical intervention. In-depth interviews explored the commonalities and differences in patient and clinician experiences of SLE and other SARDs flares and the underlying reasons for any differences.

The worsening of disease symptoms, and subsequent adverse impact on patient quality of life, was identified by both patients and clinicians as a fundamental aspect of SARD flares. In common with existing studies, most SARDs patients reported pain and fatigue during flares [7, 18]. Patients also discussed neuropsychiatric symptoms, such as cognitive dysfunction, mood and sensory changes, which have been documented in our team’s previous research [12, 19, 20]. Our interviews revealed how flares adversely impact employment, which mirrors existing literature in RA, SLE, myositis and psoriatic arthritis [18, 21–23].

A notable novel finding from this study was the discrepancy between patients’ and clinicians’ views on the speed of flare onset, with clinicians having significantly higher ratings for flares ‘*occur very gradually (in a matter of weeks or months)*’ and patients having significantly higher ratings for flares ‘*occur suddenly (in a matter of min or h)*’. To our knowledge, this aspect of flare onset has not been explored in previous studies. However, research into RA flares has identified that flares with a sudden onset may require quicker reaction and treatment compared with flares with a gradual onset [24]. Therefore, underestimation of the speed of flare onset by clinicians may impede timely and effective disease management.

Patients scored significantly higher on statements about patients being able to tell when they were flaring, the presence of warning signs of flares (‘prodromes’) and flare symptoms being similar each time. In contrast, clinicians scored significantly higher on statements about doctors being able to tell when patients were flaring and flares causing changes in clinical test results. This dichotomy was reflected in the interviews, where patients expressed frustration that it was difficult to get medical treatment for flares which did not show up by physical examination or abnormal clinical test results. Previous research into SLE flares has found similar results, with an interview-based study finding that although very few patients mentioned laboratory measures in their descriptions of flares, over half of rheumatologists interviewed required objective findings to diagnose a flare [7]. However, systematic review research has identified a lack of effective biomarkers for predicting SLE flares, suggesting that reliance on these tests over patient reports may be problematic [25]. Furthermore, patient assessment of RA flare status has been found to be accurate in detecting and ruling out clinically significant flares, indicating the important role of patients’ recognition of flares for effective disease control [26].

This study also highlighted how the lack of consensus surrounding flare recognition has repercussions for timely and effective flare management, with patients and clinicians discussing how subclinical flares are less likely to trigger the initiation of new or increased medical treatment. In the survey, clinicians rated statements relating to flares requiring new or increased medication significantly higher than patients. This may reflect a selection bias whereby clinicians see patients for flares predominantly in cases where they are seeking medical treatment. Recurrent disease flares have a major adverse impact on the long-term accumulation of tissue damage, impaired quality of life, work and social disability, increased healthcare resource use and premature mortality [27]. Prompt recognition and management of disease flares by patients and clinicians could reduce damage accumulation and improve long-term outcomes [28], yet our study suggests that many flares are currently not being reported, recognised and/or recorded. This has likely led to skewed understanding and definition of flares in the literature towards the clinicians’ perspectives and those requiring medical intervention.

In interviews, patients discussed often self-managing or self-medicating during flares. For many of our interviewees, this was not a preference but a necessity, driven by perceptions of being disbelieved or inadequately supported by clinicians who were time-poor and unwilling to prescribe new or increased medication without objective results. This is consistent with other studies finding that although medication change is associated with better flare outcomes in RA, the majority of patients changing medication do so without consulting a clinician [29]. Patient interviewees also discussed preferring to self-manage their flares because the medical treatment offered had not been effective for improving flare symptoms. This has been found in previous studies, where SLE patients preferred to self-manage by pacing and resting rather than seeking medical treatment [7]. Clinicians and patients also discussed how self-management could vary over time, with the tendency and ability for self-management increasing as patients got more experienced with their disease. Although there is limited evidence for the link between time since diagnosis and self-management, studies have found that interventions aimed at patient education may have a positive impact on self-efficacy, suggesting that better understanding of one’s disease may improve self-management [30].

Our findings raise important questions about whether generic clinician-derived flare measures can adequately capture the breadth, complexity and heterogeneity of patient flare experiences. Rather than relying solely on validated flare instruments, our findings emphasise the importance of trusting partnerships and communication between clinicians and patients. Furthermore, future flare measurement tools should be designed to be adaptable to the individual. We propose individualised ‘care and flare’ plans to be drawn up based on the patient’s own flare characteristics. This would allow individualised monitoring and management of flares based on each patient’s unique flare and symptom patterns.

### Strengths and limitations

Strengths include the multi-disciplinary team of researchers, patients and clinicians who were involved in all stages of the study. Partnership with patients led to substantial changes and improvements at each stage of the research process including less clinical wording and suggestions to focus on documenting all flares. Another strength was the mixed methodology, whereby contextual insights gained from the in-depth interviews helped us interpret the differences between clinician and patient perspectives identified quantitatively. Triangulation of quantitative and qualitative findings demonstrated a high level of concordance between the two sources. This integration provided richer and more robust evidence of the identified clinician–patient differences than either quantitative or qualitative methods in isolation.

Despite our efforts to recruit participants through a range of channels (see Supplementary Data S1), survey and interview participant groups lacked diversity, with the majority of patients being White and female. Another limitation was that clinicians were asked to rate flare statements based on the average patient, whereas patients reported on their own individual experiences. These perspectives may therefore not be directly equivalent; for example, clinicians may be less likely to select ‘*always*’ or ‘*never*’ when talking about patient averages compared with patients talking about their individual experiences. To minimise the effect of this, we have combined percentages of participants selecting either ‘*always*’ or ‘*often*’ in the description of results. The quantitative analysis focused on SLE flares only. While it was appropriate to narrow the scope to enable clinicians to provide specific and comparable answers, future research should also quantitatively examine the experiences of flares across SARDs and other autoimmune diseases. We also note the possibility of sampling bias in the clinician group, whereby clinicians who were patient-centred were more likely to participate in an interview and/or survey with no guaranteed remuneration.

## Conclusion

This study highlights key differences in how SARD patients and clinicians describe disease flares, most notably in relation to the speed of flare onset and methods for flare recognition. These differences have implications for care and the design and interpretation of clinical trials involving flares and challenge the use of biomarkers and disease activity indices for assessing flare status. Both groups agreed that flares had a considerable adverse impact on patients’ quality of life, highlighting the need the development of interventions which support patients at every stage of the flare cycle, including identifying triggers and educating about self-management. A clinical culture that validates and records patient-reported flares, even when objective evidence is limited, should be encouraged. This could enhance trust, ensure timely intervention and empower patients to manage flares effectively, in addition to facilitating research into flares encompassing the full spectrum of symptoms and patient experiences.

## Supplementary material

Supplementary material is available at *Rheumatology* online.

## Supplementary Material

keag443_Supplementary_Data

## Data Availability

We will make data available on reasonable academic request once all INSPIRE-Flare studies have been analysed.
