# Supplementary material for: Describing flares: a mixed methods study comparing patient and clinician perspectives in systemic lupus erythematosus and other rheumatic diseases
Source: Rheumatology (Oxford). 2026 Aug 20;65(9):keag443. doi: 10.1093/rheumatology/keag443 (PMC13592472; doi:10.1093/rheumatology/keag443)
Supplement: Supplementary file 1 [file keag443_supplementary_data.docx]

## **Supplementary Data S1: Additional methodological information for the INSPIRE-Flare research studies**

#### **Contents**

| **Section** | **Page number** |
| --- | --- |
| Study design | 1 |
| Recruitment | 1 |
| Survey design | 2 |
| Interviews | 2 |
| Research team and reflexivity | 3 |
| Data cleaning | 3 |
| Analysis | 4 |
| GRIPP2 checklist | 5 |
| STROBE checklist | 5 |
| INSPIRE-Flare survey section (patients) | 8 |
| Challenges in Care survey section (clinicians) | 11 |

#### **Study design**

The INSPIRE-Flare research project is a sub-study of the broader INSPIRE (Investigating Neuropsychiatric Symptom Prevalence and Impact in Rheumatology patient Experiences) project (1). It aims to explore and compare how systemic autoimmune rheumatic disease (SARD) patients and clinicians, and patients from different disease groups, describe the word “flare”.

The first INSPIRE-Flare study, comparing autoimmune patient and clinician views, will use mixed-methods to allow for in-depth exploration and explanation of the quantitative data received from the surveys. By combining quantitative and qualitative approaches, mixed methods reduce the limitations of each individual method and yield a more thorough and insightful picture of the phenomenon being explored.(2). Integration of qualitative and quantitative data can therefore provide insights beyond the information gained from one or the other (3). The second INSPIRE-Flare study, comparing autoimmune patients from different disease groups, will use quantitative methods only due to the large amount of survey data for multiple aspects of flares and disease types.

The main sources of data for INSPIRE-Flare were the INSPIRE-Flare patient survey, the Challenges in Care clinician survey and in-depth interviews with patients and clinicians.

We combined moderate realist and constructivist paradigms for qualitative research, as this allows for multiple interpretations of a shared reality (4). Through these interpretations, we can gain deeper knowledge of individual experiences and use this understanding to improve the lives of patients and others involved. This also fits with our ethos of working with participants to construct themes as they arise. We aim to be inclusive and representative of the collective patient voice, while also incorporating divergent views. The key aims of all INSPIRE-Flare studies are to represent the views and experiences of SARD patients and clinicians, to improve understanding about these diseases and communication between patients and clinicians, and to further the interests of SARD patients and clinicians. This includes ensuring participant confidentiality and ethical standards were maintained throughout the studies.

#### **Recruitment**

For the INSPIRE-Flare survey, we recruited participants by distributing the recruitment poster on social media channels, online forums, through charities and community programs. Charities and community programs were selected to access participants from a range of sociodemographic background, with the help of our Inequalities team lead, SAT. We also invited previous INSPIRE study participants who had consented to receive information about future studies via email. We also displayed the recruitment poster in rheumatology clinics across the UK and delivered posters for rheumatologists to hand out to eligible patients. NHS sites included Guys’ and St Thomas’ Hospital, London, St George’s Hospital, London and Queen Elizabeth Hospital, Birmingham. Participants were eligible if they self-reported having received an autoimmune disease diagnosis on clinical correspondence and were over 18 years old. Eligibility was confirmed at the start of the survey and ineligible participants were taken to the end of the survey and not counted (Question 4.2 in the INSPIRE-Flare patient survey).

For the Challenges in Care clinician survey, we recruited participants through social media and researchers’ professional networks and contacts. Participants were eligible if they were clinicians and treated patients with autoimmune diseases. Specialities included, but were not limited to, rheumatologists, psychiatrists, physiotherapists, general practitioners (GPs) and neurologists.

The surveys were accessed online in Qualtrics. Participants could access them via short links and QR codes on the recruitment poster and/or in the post caption. Both surveys included a consent question at the start, where patients could access the participant information sheet, confirm their understanding and provide informed consent to take part. The INSPIRE-Flare patient survey was available from April to June 2025 and the Challenges in Care clinician survey was available from December 2024 to March 2025. Both surveys included a question where participants could opt in to receive information about future studies, be part of the prize draw and/or be sent the results of the study (Q3.1 in the INSPIRE-Flare patient survey).

Alongside the Challenges in Care clinician survey, our team conducted the Challenges in Care patient survey, which asked autoimmune patients about related topics such as inequalities in care, clinician concordance and menopause experiences. The patient interviewees were recruited through emailing patient participants who had opted in to being invited to interview following their participation in the Challenges in Care and INSPIRE surveys. Similarly, clinician interviewees were recruited through emailing Challenges in Care clinician participants who had opted in to being invited to interview following their participation. Informed consent for interviews was provided verbally and audio-recorded at the start of each interview.

There were no pre-defined recruitment targets for either group of participants but our aim was to recruit as large and diverse a sample as possible. The surveys were available online and reminders were sent multiple times by charities and the study team to charities, social media, online forms and to previous participants via email. Recruitment for the INSPIRE-Flare patient survey was continued until the proportions of non-White and female participants were below 90%, with substantial efforts made to increase sample diversity.

#### **Survey design**

The INSPIRE-Flare survey and Challenges in Care surveys each featured a matrix question (Q7.3 and Q5.7 below) including statements about flares, which patients and clinicians rated on a Likert scale of “*always*”, “*often*”, “*sometimes*”, “*rarely*” and “*never*”. The statement wording was altered slightly to suit the patient/clinician group, with patients being asked about their own experiences and clinicians being asked about the average systemic lupus erythematosus (SLE) flare. Patients were also given an option for “*not sure*” but these responses were not included in the main analysis of the first INSPIRE-Flare paper (patients and clinicians) to allow for comparison with clinicians. Patients could add additional comments about what a flare meant to them in an open-text response question and 70% of patients did so.

The INSPIRE-Flare and Challenges in Care surveys were designed with input from patient partners, clinicians and rheumatology charities to ensure questions were understandable and relevant. Both surveys were assessed using the “think aloud” method with patient and clinician partners to ensure the questions were understandable and acceptable (5). Despite this testing, analysis of responses to the statement “*my autoantibodies blood tests get higher during a flare*” suggested that patients had not understood the statement. This was identified by team clinicians, who noticed that patients from diseases where existing autoantibody blood tests usually do not change with increased disease activity had responded “*always*” or *“often”.*

#### **Interviews**

Interviews were carried out before, during and after the collection of survey data. Survey participants who had opted in to being invited to interview were purposively sampled to include a range of sociodemographic characteristics, disease experiences and clinician specialities. Participants were invited to interview via email and up to two follow-up emails were sent to participants who had not responded. Approximately 55% of those contacted responded and accepted the interview invitation. Non-attendance was mainly due to participants not responding to the email, but a minority replied saying they were too busy or too unwell to participate in an interview. The majority of interviews took place online, through Microsoft Teams or Zoom, with a small number being via telephone if participants preferred.

There were two semi-structured interview schedules, one for clinicians and one for patients. Both schedules included questions about participants’ understanding of the word “*flare*”, how flares could be identified, what triggered and prevented flares, which symptoms occurred just before flares and whether flares required medical treatment. Patients were also asked whether their disease flared and how flares affected their lives. Clinicians were asked about the support they could offer for flares and how flares differed between disease groups. Interviewees were not given access to the interview questions beforehand or the transcripts afterwards. Interviewers used open-ended questions initially and then tailored subsequent questions depending on the participants’ experiences and priorities for discussion, as long as they were in the remit of the study.

Interviewers were researchers at the University of Cambridge (MS, AT, MP, ST), a master’s student at Kings College London (UP) and a medical student at the University of Cambridge (RL). All interviewers were female, but there was variety in interviewer age, ethnicity and seniority. Researchers were already experienced in qualitative research methods and students received a 6-week training programme and regular feedback and support from the study principal investigator (MS) and their research mentor. Interviewers did not disclose any characteristics to the participants other than those visible on video conferencing software. Interviewers had no relationship with participants before interviewing, except for some clinician participants who were known to the study team. Interviewers reflected on their own biases, expectations and experiences and how this influenced their interview style and interpretation of the data. Interviewer bias was managed by ensuring all questions were clear and open-ended and through discussions with the multi-disciplinary team.

#### **Research team and reflexivity**

Our multi-disciplinary research team consists of researchers, patients, rheumatologists, psychiatrists, neurologists, statisticians and data managers.

Individual INSPIRE-Flare papers will be co-authored by different groupings of team members depending on interests, availability and health.

Patient and clinician research partners were involved as equal members of our research team in every stage of this study in order to increase the relevance and impact of our findings. Our research partners included three SARDs patients, two neurologists, five rheumatologists, two psychiatrists and one medical student who was also diagnosed with a SARD. All research partners were involved with developing the topic guide and surveys, revising the themes and subthemes and presenting the findings. The perspectives of and feedback from patient and clinician members of the research team sometimes differed, such as in how we interpreted the findings. In these cases, we discussed key points in multi-disciplinary team meetings, anonymising opinions where preferred, to facilitate team discussion and create solutions which all co-authors were satisfied with.

Patient and clinician research partners were sometimes unable to attend team meetings where we discussed aspects of the study, due to conflicting schedules or periods of ill health. Although we provided minutes from all meetings and requested research partners’ opinions via email, some research partners were not involved in some phases of the study development, execution and write up.

Clinicians and patients involved in research may have had varying degrees of conscious and unconscious bias surrounding the topics investigated. The potential for any individual team member bias influencing the study was mitigated by the diverse multi-disciplinary study team providing thorough and multi-perspective ongoing feedback on the data analysis, reflecting on potential bias in themselves and identifying possible bias in each other. Patient representatives play a major role in all our studies and were initially selected to be study team members based on having a combination of positive and negative medical experiences, all demonstrating an objectivity necessary for active involvement as collaborators in this research. Rheumatology charity staff were consulted on their opinions and added depth of understanding of these patient groups.

#### **Data cleaning**

Data cleaning excluded participants without autoimmune diseases and those clinicians and patients who had not completed up to the flare matrix of the surveys. Disease groups with less than 30 participants were combined where applicable to ensure sufficient numbers for statistical comparison. For example, for the sub-study including non SARD autoimmune diseases, alopecia and vitiligo (and others) were combined into “autoimmune dermatological”. Rare autoimmune diseases which did not medically fit into a wider group were combined into “other autoimmune”. Autoimmune diseases which were considered by team clinicians to not have a flare pattern (e.g. T1D and Autoimmune Encephalitis) were excluded from this part of the study.

#### **Analysis**

*Qualitative (INSPIRE-Flare patient and clinician paper only)*

Interview data was analysed using reflexive thematic analysis, with themes developed directly from the transcript data (6). Open-ended survey responses were checked to confirm the findings in the themes and subthemes. The number of interviews was based on the model of informational power, which considers the research question, heterogeneity of participants and richness, depth and relevance of data (7).

The stages of thematic analysis involved an inductive-deductive process:

1) Immersion in the data whereby interviewers frequently read each full transcript throughout the stages of interviewing, analysis and write-up. Researchers used mind-maps, spreadsheets and other tools to understand the key topics and themes in the data. Transcripts and extracts were also read and discussed by multiple team members to improve reliability and ensure multiple possible interpretations of the data were represented. Lead researchers also reflected on their own biases and experiences which may influence their interpretation of the data to minimise the impact of subconscious bias on analysis.
2) Coding the data in which a coding scheme is developed based on the interview transcripts. Transcripts were coded line-by-line using NVivo 14 software. Some sections received multiple codes if they related to multiple topics or themes in the data. The same researcher coded all transcripts so that they were able to identify common themes across multiple interviewees.
3) Refining and re-coding the data through discussions with the wider research team. The lead researcher presented their initial codes with illustrative quotes to other researchers, patients and clinicians on the research team. The team gave feedback and discussed how the codes might fit together thematically. Following discussion, the lead researcher adapted the codes and re-coded where necessary according to the team’s feedback.
4) Identification of themes and subthemes by combining related codes into broad categories. Initial themes and subthemes were generated by the lead researcher and then presented to the wider team for feedback. Themes and subthemes were refined and agreed by the team.

#### Quantitative

Survey data was transferred from Qualtrics to Excel and SPSS for analysis. Our data manager, JB, anonymised and password protected all data files, and stored them on the University of Cambridge secure data platform.

Data was presented graphically and descriptively to ensure accessibility for a diverse range of readers. Descriptive statistics (frequencies, percentages, mean, standard deviation, standard error, median and interquartile range) were used as appropriate to describe the data. Analysis involved linear regression, correlation and comparison of means and frequencies. T-tests were used for comparing the means of two groups and ANOVA for multiple groups.

We have sometimes reported percentages of grouped responses, such as “*always*” and “*often*”, to convey general opinions. However, statistical significance and reported p-values are calculated from all categories of response. We investigated the association between the groups of interest (patients from different disease groups and clinicians) and the categorical outcomes using chi-square tests. For some analyses, we had to group smaller demographic groups in with larger ones to achieve sufficient numbers for statistical analysis. For example, we grouped trans females into ‘female’, trans males into ‘male’. Statistical significance was p<0.05 and 95% confidence intervals were used. Bonferroni corrections were applied appropriately.

The surveys were long and had varying levels of attrition at each survey stage. Therefore, we analysed each question separately using the number of participants completing that section rather than the number who commenced the survey. The online survey forced responses to most questions, with a “*not sure*” or “*prefer not to say*” option given as appropriate. This design ensured that there was no missing within-section data.

Qualitative and quantitative methods were considered together and integrated at every stage of the process including design, data collection, analysis and write up.

#### **GRIPP2 checklist short form – INSPIRE-Flare paper 1 (patients and clinicians)**

| **Section and topic** | **Item** | **Reported on page number** |
| --- | --- | --- |
| Aim | Report the aim of PPI in the study | Methods, page 5 of main manuscript |
| Methods | Provide a clear description of the methods used for PPI in the study | Research team, pages 4 and 5 of supplementary information |
| Study results | Outcomes—Report the results of PPI in the study, including both positive and negative outcomes | Research team, pages 4 and 5 of supplementary information |
| Discussion and conclusions | Outcomes—Comment on the extent to which PPI influenced the study overall. Describe positive and negative effects | Research team, pages 4 and 5 of supplementary information |
| Reflections/critical perspective | Comment critically on the study, reflecting on the things that went well and those that did not, so others can learn from this experience | Research team, pages 4 and 5 of supplementary information |

#### **STROBE checklist**

| **No** | **Content** | **Recommendation** | **Actioned** |
| --- | --- | --- | --- |
| 1 | Title and abstract | (a) Indicate the study’s design with a commonly used term in the title or the abstract   (*b*) Provide in the abstract an informative and balanced summary of what was done and what was found | Yes, in the title  Yes, in the abstract |
| 2 | Introduction Background/rationale | Explain the scientific background and rationale for the investigation being reported | Yes, in the introduction |
| 3 | Objectives | State specific objectives, including any prespecified hypotheses | Yes, in the methods |
| 4 | Methods – study design | Present key elements of study design early in the paper | Yes, in the main text and supplementary material |
| 5 | Methods - setting | Describe the setting, locations, and relevant dates, including periods of recruitment, exposure, follow-up, and data collection | Yes, in the main text and supplementary material |
| 6 | Participants | Give the eligibility criteria, and the sources and methods of selection of participants | Yes, in the main text and supplementary material |
| 7 | Variables | Clearly define all outcomes, exposures, predictors, potential confounders, and effect modifiers. Give diagnostic criteria, if applicable | Yes, in the main text and supplementary material |
| 8 | Data sources/ measurement | For each variable of interest, give sources of data and details of methods of assessment (measurement). Describe comparability of assessment methods if there is more than one group | Yes, in the main text and supplementary material |
| 9 | Bias | Describe any efforts to address potential sources of bias | Yes, in the main text and supplementary material |
| 10 | Study size | Explain how the study size was arrived at | Yes, in the supplementary material |
| 11 | Quantitative variables | Explain how quantitative variables were handled in the analyses. If applicable, describe which groupings were chosen and why | Yes, in the supplementary material |
| 12 | Statistical methods | (a) Describe all statistical methods, including those used to control for confounding (b) Describe any methods used to examine subgroups and interactions (c) Explain how missing data were addressed (d) If applicable, describe analytical methods taking account of sampling strategy (e) Describe any sensitivity analysis | (a) and (b) in main text and supplementary material.  (c) in supplementary material  (d) not applicable for this study  (e) in supplementary material |
| 13 | Results | (a) Report numbers of individuals at each stage of study—eg numbers potentially eligible, examined for eligibility, confirmed eligible, included in the study, completing follow-up, and analysed (b) Give reasons for non-participation at each stage (c) Consider use of a flow diagram | (a) and (b) in main text and supplementary material  (c) not appropriate for this study |
| 14 | Descriptive data | (a) Give characteristics of study participants (eg demographic, clinical, social) and information on exposures and potential confounders | Yes, in main text and supplementary material |
|  |  | (b) Indicate number of participants with missing data for each variable of interest | Yes, in the main text graphs and participant table |
| 15 | Outcome data | Report numbers of outcome events or summary measures | Yes, in main text |
| 16 | Main results | (a) Give unadjusted estimates and, if applicable, confounder-adjusted estimates and their precision (eg, 95% confidence interval). Make clear which confounders were adjusted for and why they were included (b) Report category boundaries when continuous variables were categorized (c) If relevant, consider translating estimates of relative risk into absolute risk for a meaningful time period | (a) and (b) TBD in main text  (c ) not appropriate for this study |
| 17 | Other analyses | Report other analyses done—eg analyses of subgroups and interactions, and sensitivity analyses | N/A |
| 18 | Discussion  Key results | Summarise key results with reference to study objectives | Yes, in results section |
| 19 | Limitations | Discuss limitations of the study, taking into account sources of potential bias or imprecision. Discuss both direction and magnitude of any potential bias | Yes, in main text supplementary material |
| 20 | Interpretations | Give a cautious overall interpretation of results considering objectives, limitations, multiplicity of analyses, results from similar studies, and other relevant evidence | Yes, in discussion |
| 21 | Generalisability | Discuss the generalisability (external validity) of the study results | Yes, in discussion |
| 22 | Other information | Give the source of funding and the role of the funders for the present study and, if applicable, for the original study on which the present article is based | Yes, in main text |

#### **INSPIRE-Flare survey** **section (patients)**

Q7.3 How often are the following statements true, thinking about your own experiences of flares?

|  | Always true for my flares | Often true for my flares | Sometimes true for my flares | Rarely true for my flares | Never true for my flares | Not sure |
| --- | --- | --- | --- | --- | --- | --- |
| Flares involve the emergence of new disease symptoms |  |  |  |  |  |  |
| Flares occur after a period of disease stability or remission |  |  |  |  |  |  |
| Flares involve changes in clinical test results (e.g., blood or urine tests) |  |  |  |  |  |  |
| Flares involve a worsening of disease symptoms compared to when I'm well |  |  |  |  |  |  |
| Flares start suddenly (in a matter of minutes or hours) |  |  |  |  |  |  |
| Flares start gradually (in a matter of days) |  |  |  |  |  |  |
| Flares start very gradually (in a matter of weeks or months) |  |  |  |  |  |  |
| Flares last for a matter of days |  |  |  |  |  |  |
| Flares last for a matter of weeks |  |  |  |  |  |  |
| Flares last for a matter of months |  |  |  |  |  |  |
| Flare symptoms are similar each time |  |  |  |  |  |  |
| Flares require an increased dose of prescribed medication (independent of whether you receive it) |  |  |  |  |  |  |
| Flares require prescription of new medication (independent of whether you receive it) |  |  |  |  |  |  |
| Flare symptoms affect my quality of life |  |  |  |  |  |  |
| I know when I am flaring |  |  |  |  |  |  |
| Doctors can tell if I am flaring by physical examination |  |  |  |  |  |  |
| My autoantibodies blood tests get higher in a flare |  |  |  |  |  |  |
| There are symptoms/signs that come before a flare, which are a 'bad sign' that a flare is coming (this is known as a prodrome) |  |  |  |  |  |  |

Q7.4 Please add any other comments about what a 'flare' means to you.

________________________________________________________________

________________________________________________________________

________________________________________________________________

________________________________________________________________

________________________________________________________________

End of Block: Defining Flares YES

#### **Challenges in Care survey section (clinicians)**

Q5.7 When thinking about flares experienced by lupus patients, how often do you think the following statements are true?  Note: We are looking for your personal opinion, there are no right or wrong answers:

|  | Never true for flares | Rarely true for flares | Sometimes true for flares | Often true for flares | Always true for flares |
| --- | --- | --- | --- | --- | --- |
| Flares involve the emergence of new disease symptoms |  |  |  |  |  |
| Flares occur after a period of disease stability or remission |  |  |  |  |  |
| Flares involve changes in clinical test results (e.g. blood or urine tests) |  |  |  |  |  |
| Flares involve a worsening of disease symptoms compared to when the patient is well |  |  |  |  |  |
| Flares start suddenly (in a matter of minutes or hours) |  |  |  |  |  |
| Flares start gradually (in a matter of days) |  |  |  |  |  |
| Flares start very gradually (in a matter of weeks or months) |  |  |  |  |  |
| Flares last for a matter of days |  |  |  |  |  |
| Flares last for a matter of weeks |  |  |  |  |  |
| Flares last for a matter of months |  |  |  |  |  |
| Flare symptoms are similar each time for the same patient |  |  |  |  |  |
| Flares require an increased dose of prescribed medication |  |  |  |  |  |
| Flares require prescription of new medication |  |  |  |  |  |
| Flare symptoms affect patient quality of life |  |  |  |  |  |
| A patient will know if their lupus is flaring |  |  |  |  |  |
| The patient's clinician will be able to tell if the lupus is flaring by physical examination |  |  |  |  |  |
| Autoantibodies increase in a flare |  |  |  |  |  |
| The patient will have symptoms that are a "prodrome" before a flare that indicates the imminent onset of a flare |  |  |  |  |  |

#### **Bibliography**

1.	Sloan M, Wincup C, Harwood R, Pollak TA, Massou E, Bosley M, et al. Prevalence and identification of neuropsychiatric symptoms in systemic autoimmune rheumatic diseases: an international mixed methods study. Rheumatol Oxf Engl. 2023 July 26;63(5):1259–72.

2.	SAGE Publications Ltd [Internet]. 2025 [cited 2025 Aug 28]. Research Design. Available from: https://uk.sagepub.com/en-gb/eur/research-design/book270550

3.	Fetters MD, Freshwater D. The 1 + 1 = 3 Integration Challenge. J Mix Methods Res. 2015 Apr 1;9(2):115–7.

4.	Amineh RJ, Asl HD. Review of Constructivism and Social Constructivism. 2015;

5.	Eccles DW, Arsal G. The think aloud method: what is it and how do I use it? Qual Res Sport Exerc Health. 2017 Aug 8;9(4):514–31.

6.	Braun V, and Clarke V. Reflecting on reflexive thematic analysis. Qual Res Sport Exerc Health. 2019 Aug 8;11(4):589–97.

7.	Malterud K, Siersma VD, Guassora AD. Sample Size in Qualitative Interview Studies: Guided by Information Power. Qual Health Res. 2016 Nov;26(13):1753–60.
